# Development of a multiplex microsphere immunoassay for the detection of antibodies against highly pathogenic viruses in human and animal serum samples

**DOI:** 10.1371/journal.pntd.0008699

**Published:** 2020-10-23

**Authors:** Rebecca Surtees, Daniel Stern, Katharina Ahrens, Nicole Kromarek, Angelika Lander, Petra Kreher, Sabrina Weiss, Roger Hewson, Emma K. Punch, John N. Barr, Peter T. Witkowski, Emmanuel Couacy-Hymann, Andrea Marzi, Brigitte G. Dorner, Andreas Kurth

**Affiliations:** 1 Biosafety Level-4 Laboratory, Centre for Biological Threats and Special Pathogens, Robert Koch Institute, Berlin, Germany; 2 Biological Toxins, Centre for Biological Threats and Special Pathogens, Robert Koch Institute, Berlin, Germany; 3 Institute of Virology, Charité –Universitätsmedizin Berlin, corporate member of Freie Universität Berlin, Humboldt-Universität zu Berlin, and Berlin Institute of Health, Germany; 4 Virology and Pathogenesis Group, National Infection Service, Public Health England, Porton Down, United Kingdom; 5 School of Molecular and Cellular Biology, Faculty of Biological Sciences, University of Leeds, United Kingdom; 6 Laboratoire National d'Appui au Développement Agricole, Bingerville, Ivory Coast; 7 Laboratory of Virology, Division of Intramural Research, National Institute of Allergy and Infectious Diseases, National Institutes of Health, Hamilton, MT, United States of America; CDC, UNITED STATES

## Abstract

Surveillance of highly pathogenic viruses circulating in both human and animal populations is crucial to unveil endemic infections and potential zoonotic reservoirs. Monitoring the burden of disease by serological assay could be used as an early warning system for imminent outbreaks as an increased seroprevalance often precedes larger outbreaks. However, the multitude of highly pathogenic viruses necessitates the need to identify specific antibodies against several targets from both humans as well as from potential reservoir animals such as bats. In order to address this, we have developed a broadly reactive multiplex microsphere immunoassay (MMIA) for the detection of antibodies against several highly pathogenic viruses from both humans and animals. To this aim, nucleoproteins (NP) of Ebola virus (EBOV), Marburg virus (MARV) and nucleocapsid proteins (NP) of Crimean-Congo haemorrhagic fever virus, Rift Valley fever virus and Dobrava-Belgrade hantavirus were employed in a 5-plex assay for IgG detection. After optimisation, specific binding to each respective NP was shown by testing sera from humans and non-human primates with known infection status. The usefulness of our assay for serosurveillance was shown by determining the immune response against the NP antigens in a panel of 129 human serum samples collected in Guinea between 2011 and 2012 in comparison to a panel of 88 sera from the German blood bank. We found good agreement between our MMIA and commercial or in-house reference methods by ELISA or IIFT with statistically significant higher binding to both EBOV NP and MARV NP coupled microspheres in the Guinea panel. Finally, the MMIA was successfully adapted to detect antibodies from bats that had been inoculated with EBOV- and MARV- virus-like particles, highlighting the versatility of this technique and potentially enabling the monitoring of wildlife as well as human populations with this assay. We were thus able to develop and validate a sensitive and broadly reactive high-throughput serological assay which could be used as a screening tool to detect antibodies against several highly pathogenic viruses.

## Author summary

Zoonotic pathogens, which are transmitted from their animal, arthropod or insect host to humans, have been responsible for some of the most devastating disease outbreaks in human history, including the largest Ebola virus disease outbreak in West Africa in 2014–2016, which resulted in over 28,000 infected persons and caused 11,310 deaths. Changes in land use practices, the climate and overall globalization concurrently brings human populations into closer contact with animals, arthropods, insects and the zoonotic pathogens they host, whilst increasing the possibility for local outbreaks to expand and spread internationally. Here we developed a multiplex serological assay that can be used to simultaneously detect IgG antibodies to 5 highly pathogenic zoonotic viruses, Ebola -, Marburg -, Crimean Congo haemorrhagic fever—, Dobrava-Belgrade- and Rift Valley fever virus. Our assay was designed to be able to detect antibodies to multiple species of viruses within each family, and was adapted to detect animal (specifically bat) as well as human antibodies. This assay could be used as a serological surveillance tool to monitor evidence of infection with several highly pathogenic viruses in human and wildlife populations, which is important for risk assessment and prevention of zoonotic human infectious disease outbreaks.

## Introduction

Multiple genera and species of viruses within the *Filoviridae*, *Nairoviridae*, *Hantaviridae* and *Phenuiviridae* families are highly pathogenic to humans, and members of these virus families have also been documented on the African continent. Several strains of *Ebolavirus* and *Marburgvirus* within the *Filoviridae* family have been known to cause sporadic disease outbreaks in multiple African countries, including the largest Ebola virus (EBOV) epidemic in West Africa in 2014–2016 which resulted in over 28,000 infected individuals and caused 11,310 deaths [1–3]. Indeed, the current EBOV outbreak in the Democratic Republic of the Congo (3,453 confirmed and probable Ebola virus disease cases and 2264 confirmed and probable Ebola virus deaths as of 24.03.2020 [4]) corroborates the on-going threat posed by ebolaviruses and highlights the need for continuous surveillance in endemic countries, as outbreaks can be extremely difficult to control. The tick-borne Crimean-Congo haemorrhagic fever virus (CCHFV), a member of the *Nairoviridae* family, has been responsible for the most cases of human morbidity and mortality, and has been detected in multiple African countries, frequently causing nosocomial infections / outbreaks [5–7]. The mosquito-transmitted Rift Valley fever virus (RVFV), which belongs to the *Phenuiviridae* family, has also been responsible for several large outbreaks of disease in Africa, resulting in thousands of human infections and deaths, in addition to economically devastating livestock losses [8–10]. Disease outbreaks of RVFV correlate strongly with environmental factors such as heavy rainfall and flooding [11]. Viruses within the *Hantaviridae* family have only recently begun to be documented in Africa, including Sangassou virus, Tanganya virus and Magboi virus, although it is speculated that there might be more as-yet-undocumented African hantaviruses [12–16].

All virus species included in this study are zoonotic, and reservoirs have been identified for all species except for ebolaviruses for which bats are the presumptive reservoir hosts [17,18]. For some of these viruses sentinel species can provide advanced warning that nearby human populations could be at risk [19–21]. Abortion storms in sheep and goats can indicate that RVFV is circulating in these animals, and unusual numbers of deceased non-human primates (NHPs) (e.g. gorillas and chimpanzees) could be a result of ebolavirus epizootics [22,23]. Transmission of these highly pathogenic viruses to humans occurs through contact with infected body fluids or excretions of their reservoir hosts, and in the case of RVFV and CCHFV also through the bite of their infected arthropod vectors (mosquitoes and ticks respectively). EBOV, MARV, and CCHFV can also be transmitted from human to human by contact with blood and other body fluids, potentially expanding exposed individual cases into epidemic outbreaks that have the potential to spread globally.

Early detection and diagnosis is paramount to preventing the transmission and spread of highly pathogenic viruses within human populations. Knowledge of their circulation in specific regions and/ or populations can ensure their inclusion in differential diagnosis of unknown disease aetiologies where appropriate. Active serological surveillance in animal populations can help to anticipate spill overs to humans, as well as aiding in the search for virus reservoirs [24,25]. Regular serological screening of livestock as sentinels for these viruses (primarily for CCHFV and RVFV) can help protect humans with whom they have regular contact from zoonotic infection, and aid in maintaining livestock health (in the case of RVFV). Most laboratory tests for serological surveillance are performed in a single test format using conventional plate-bound enzyme linked immunosorbent assay (ELISA) techniques. It is often desirable, however, to monitor the serostatus against different viral diseases simultaneously in order to reduce materials used, as well as cost and effort. Among the multiplex platforms described the suspension array technology, also called Luminex xMAP technology (Luminex Corp., Austin, USA), is of interest for broad-range use in standard laboratories, as it is commercially available and technically open to different applications [26]. The suspension array technology uses magnetic polystyrene microspheres with a diameter of 6.5 μm which are embedded with precise ratios of red and infrared fluorescent dyes, thus yielding an array of 100 bead sets which are spectrally unique and can be distinguished by flow cytometry. In a serological assay, the assay principle is similar to an indirect ELISA: First, differently coloured microspheres are covalently coupled to immunodominant antigens of different viruses (e.g., either purified recombinant proteins or cell lysates of virally infected cells); individual serum samples are then incubated with a mixture of microbeads coupled to different immunodominant antigens and positive signals are detected using biotinylated anti-species antibodies followed by the fluorescent reporter streptavidin-phycoerythrin. The Luminex flow cytometer addresses (i) the fluorescent signature of the microsphere set and (ii) the intensity of the reporter signal per microsphere set. Thus, antibodies against multiple viruses can be detected simultaneously from a minimal sample volume of 1–2 μL serum.

Here, we describe the development of a broadly reactive multiplex microsphere immunoassay (MMIA) for the detection of antibodies in human and animal serum samples as generated in response to infection by members of the *Filoviridae*, *Phenuiviridae*, *Nairoviridae* and *Hantaviridae* families.

The nucleoprotein (NP) of EBOV and MARV and the nucleocapsid proteins (NP) of CCHFV, Dobrava-Belgrade virus (DOBV) and RVFV were chosen to function as the target antigens in our MMIA as NPs have previously been shown to possess high immunogenicity, in addition to their sequences being conserved across different virus species of one genus [27–35]. Therefore, by using these proteins each antigen in the MMIA is designed to be broadly reactive, and should be able to detect antibodies to multiple virus species within each family (with the possible exception of the new-world hantaviruses).

Following assay optimisation utilizing hyperimmune animal serum for individual viruses, the MMIA was tested for specificity and validated with the serum of laboratory-infected NHPs (for EBOV, MARV and RVFV) and previously characterized human serum (for CCHFV and DOBV). Following validation, the MMIA was used to screen 129 human serum samples collected from Guinea in 2011 and 2012. Furthermore, a pan-species antibody detection system was established in order to broaden the scope of the newly established assay so as to enable versatile serosurveillance. This was achieved by substituting the secondary antibody with a mixture of biotinylated protein A and protein G. The assay was then validated for its ability to detect antibodies from other animal species using the serum of insectivorous micro bats caught in the Ivory Coast and then immunized with EBOV- and MARV- virus-like particles (VLPs). Here, we present the development of a high-throughput screening tool that can be used to assess the presence and prevalence of IgG antibodies against 5 different highly pathogenic viruses in human and animal populations. Our MMIA was used to successfully detect antibodies against these viruses in a cohort of human serum samples from Guinea, the German blood bank and wild bats from the Ivory Coast, proving its versatility as a broadly reactive surveillance tool.

## Materials and methods

### Ethics statement: animal and human sera

#### Hyperimmune sera against EBOV NP, MARV NP, RVFV NP and CCHFV NP

In order to establish the assay by optimising the coupling of NPs to microspheres and assessing cross reactivity between antigens, hyper immune mouse serum was generated against EBOV NP (Zaire ebolavirus, isolate Ebola virus/H.sapiens-tc/COD/1976/Yambuku-Mayinga), MARV NP (Lake Victoria marburgvirus—Angola2005, strain "Ang0998") and RVFV NP (isolate "688/78") using the recombinant proteins. All animal experiments with mice using bacterially expressed EBOV NP, MARV NP, and RVFV NP were registered and approved by the responsible governmental authorities (Office for Health and Social Affairs Berlin, LAGeSo; registration number H0129/19). Animals were housed according to national regulations. The physical condition of the animals was monitored daily. No animal became ill prior to the experimental endpoint. Hyperimmune sera were generated through serial inoculation of mice with the purified recombinant NPs of RVFV, EBOV and MARV according to standard procedures [26]. Hyperimmune sheep serum containing anti-CCHFV NP antibodies was generated by AltaBiosciences Ltd. (United Kingdom) using 1 mg purified CCHFV NP (highly similar to strain Baghdad 12) in a 1-ml volume [36,37].

#### Human and non-human primate serum samples

Human serum samples that had previously tested serologically positive for DOBV were kindly provided by J. Hofmann from the Institute of Virology, Charité –Universitätsmedizin Berlin, Berlin, Germany. The 129 human serum samples collected from febrile patients in Guinea in 2011 and 2012 were collected in accordance with (ethics statement 09/CNERS/12). Anonymised human serum samples from the German blood bank (n = 88), the Robert Koch Institute (n = 3) and the Laboratoire National d'Appui au Développement Agricole (n = 2) were used to optimise the MMIA and as a presumptive “negative control” population in order to establish background readings for the MMIA as well as the recombinant NP based EBOV and MARV in-house ELISAs. All human sera were heat inactivated at 56°C for at least 30 minutes, prior to any serological analysis.

In order to validate the MMIA for anti CCHFV IgG antibody detection, human serum samples (10) were obtained from the WHO Collaborating Centre for Virus Research & Reference (Special Pathogens)–PHE Porton Down, UK. Original samples were collected from febrile patients who contracted disease in (i) Tajikistan in 2015–2016, (ii) Nigeria in 2015 and received a laboratory diagnosis of CCHFV. Sera samples were not collected specifically for this work, thus ethical approval for the study design was not required. Samples were anonymised within Tajikistan and Nigeria and investigators were supplied with sequentially numbered samples. Samples received a confirmatory test at Public Health England, UK.

Serum samples from NHPs infected with RVFV (strain not specified), MARV or EBOV were used as surrogate serum samples to validate the MMIA as known serologically positive human samples for these viruses were not available. NHP sera have previously been shown to be able to function as a substitute for human sera in serological assays [38]. NHP sera positive for antibodies against RVFV (strain not specified), CCHFV (strain not specified) and EBOV were kindly provided by Gary Kobinger of the National Microbiology Laboratory, Winnipeg, Canada (animal protocol number H-08-015, title: Development of antiserum against for Machupo, Junin, CCHFV, Hendra, Nipah, and Rift Valley Fever Virus in non-human primates for human diagnostic purposes) to function as positive controls in the MMIA. Sera used for validation of the MMIA were generated as part of various vaccine trials through the infection of NHPs with EBOV-Mayinga [39], EBOV-Makona [40,41], Reston virus (RESTV Pennsylvania 1989 and Philippines 2008) and MARV-Angola in the BSL-4 facility at the NIH Rocky Mountain laboratories (RML), Montana, USA. Serum samples were taken at various time points post-infection and used to validate the MMIA for reactivity to the corresponding NP coupled microspheres. All NHP sera were inactivated on dry ice by gamma irradiation according to IBC-approved standard operating procedures prior to any manipulation outside of a BSL-4 laboratory.

#### Bat serum samples

Twenty *Mops condylurus* bats were captured with mist nets at a residence in Koffikro Village in Ivory Coast (geographic coordinates: N 05° 19.340´; W 003° 49.431´). Subsequently, the bats were kept in captivity at LANADA Institute (Laboratoire National d’Appui au Développement Agricole) Bingerville, Ivory Coast. Capture, animal work and immunization was executed in accordance with ethics agreement by the respective governmental authority (LANADA Institute, No. 05/virology/2016). The bats were pre-bled, followed by first subcutaneous inoculation and boosted after 15 days with 100 μl VLPs-adjuvant formulation into the loose skin on the neck. Appropriate amounts of EBOV-VLPs (#0550–001, IBT Bioservices, 1.54 mg/ml) or MARV-VLPs (#0566–001, IBT Bioservices, 3.725 mg/ml) were diluted in sterile PBS to a final concentration of 50 μg of VLPs (10 bats each for EBOV- and MARV-VLPs) in 50 μl and mixed with 50 μl Alhydrogel adjuvant 2% (CAS No 21645-51-2, InvivoGen). Serum was collected 24 days after first inoculation by cardiac puncture under anaesthesia with Isoflurane (1214, cp-pharma) and frozen at -80°C.

### Recombinant nucleo/nucleocapsid proteins

The nucleo and nucleocapsid proteins (NPs) of EBOV, MARV, CCHFV, RVFV and DOBV were chosen as the antigens in the MMIA, due to their high immunogenicity and because their sequences are conserved across different virus species enabling the detection of antibodies against multiple virus species within one family. To generate recombinant NPs full length NPs of RVFV (GenBank accession DQ924959), EBOV (NP_066243), MARV (ABE27061), CCHFV (unpublished NP sequence with three conserved amino-acid substitutions (T111I, R195H and H445D) from the published Baghdad 12 strain (GenBank accession CAD61342.1)) and DOBV (GenBank accession. AJ269550.1) were bacterially expressed as His-tagged fusion proteins and purified using Ni-NTA affinity chromatography. The plasmids encoding EBOV NP, MARV NP and RVFV NP were synthesised, and the recombinant proteins were custom-made by Genexpress (Berlin, Germany). Briefly, codon-optimized genes, for expression in *E*.*coli*, were cloned into expression vector pQE100S. Expression was induced with 2 mM isopropyl β-D-1-thiogalactopyrano-side overnight at room temperature, His-tagged proteins were isolated under native (Protein X) conditions and purified using Profinity Ni-IMAC Resin column (BioRad, California, US) according to standard procedures. The bacterial lysate used to coat one microsphere region and to pre-incubate the serum samples was also supplied by GenExpress and was generated by transforming the empty expression vector pQE100S into *E*.*coli*. The culture was grown after induction for 4 hours at 37°C and the bacterial lysate was then also isolated under native conditions. CCHFV NP and DOBV NP were expressed and purified as previously described [42,43]. Protein gel electrophoresis followed by Coomassie staining was utilized to assess the purity of each protein (S1 Fig). His-tagged or tag-free (CCHFV only, where His-tag had been removed by cleavage and size exclusion chromatography) proteins were subsequently coupled to paramagnetic Magplex microspheres.

### Multiplex microsphere immunoassay

#### Protein coupling to microspheres

All proteins were dialysed into PBS and then coupled covalently to different regions (regions 12, 14, 15, 18, 20, 21 and 25) of Magplex microspheres (Luminex, Austin, Texas, USA) using the standard protocol as outlined in the xMAP Cookbook (44). Briefly, 1 × 10^6^ microspheres were washed in distilled water and subsequently resuspended in NaH_2_PO_4_ prior to activation by addition of Sulpho-NHS (24520, Thermo Fisher Scientific) and EDC (22980, Thermo Fisher Scientific). Microspheres were then washed and resuspended in 50 mM MES buffer pH 5.0 (or PBS pH 7.2 in the case of EBOV NP and MARV NP) prior to the addition of varying amounts (5–70 μg) of either EBOV NP (region 20), MARV NP (region 18), RVFV NP (region 15), CCHFV NP (region 12), and DOBV NP (region 14), as well as 50 μg bacterial lysate proteins (region 21) and buffer without any proteins (region 25) that were used as negative controls. Bacterial lysate (derived from *E*.*coli* transformed with an empty expression vector) coupled to microspheres, as well as activated, un-coupled microspheres were included in every assay (every microsphere mix) to control for antibodies binding non-specifically to bacterial proteins or the microspheres themselves. Proteins were incubated with microspheres for 4 hours at room temperature with rotation, after which protein-coupled microspheres were washed twice in PBS-TBN (PBS, 0.1% BSA, 0.1% Tween-20 and 0.05% sodium azide) and then stored in PBS-TBN at 4°C.

#### Assay optimisation and detection of human IgG antibodies

The MMIA assay was optimised by varying the amount of each NP coupled to microspheres, the concentration of serum, the assay buffer, and the percentage of *E*.*coli* lysate used in the assay buffer in the pre-incubation step. The final optimised assay conditions and protocol are as follows: Serum samples were diluted 1:400 in optimised assay buffer (LowCross buffer (100 125, Candor) + 5% *E*.*coli* lysate) and incubated for 30 minutes at room temperature prior to incubation with NP coupled microspheres. Each diluted serum sample was then added in triplicate to a mix of 7 microsphere regions (5 microsphere regions bound to viral proteins plus 2 control regions), containing 1,000 microspheres of each region. The serum-microsphere mix was then incubated for 1 hour at room temperature with continuous shaking (700 rpm). The microspheres were subsequently washed 3 times in wash buffer (0.1% Tween-20 in PBS pH 7.2) prior to incubation for 30 minutes in biotin-conjugated goat anti-human IgG secondary antibody (Fc-gamma specific, 109-065-088, Dianova (Jackson)), diluted 1:2,500 in LowCross buffer. Microspheres were washed again 3 times in wash buffer and then incubated for 30 minutes in Streptavidin-R-Phycoerythrin (PJRS27, Prozyme) diluted 1:2,500 in LowCross buffer, with continuous shaking. A final wash step (3 × in wash buffer) preceded the re-suspension of the antibody-bound microsphere mix in PBS containing 1% BSA and analysis on a Bio-Plex 200 (Bio-Rad Laboratories, Munich, Germany). Care was taken to keep the fluorescent Magplex microspheres protected from light at all times.

#### Detection of bat sera in pan-species MMIA

The MMIA was adapted to detect specific antibodies targeting EBOV NP and MARV NP from bat serum samples by using a mixture of biotinylated protein A and protein G (29989, Pierce Protein A, Biotinylated, and 29988, Pierce Recombinant Protein G, Biotinylated Thermo Fisher Scientific). The MMIA was carried out as described above, with the exception that biotinylated Protein A and Protein G were diluted to a final concentration of 2 μg/mL each in assay buffer and used in place of the biotinylated anti-human IgG secondary antibody, and a 1:200 dilution of bat serum was used instead of a 1:400 dilution, as this resulted in a higher signal to background ratio.

### Confirmation assays

Further testing of serum samples was performed by re-testing selected serum samples (including those that tested positive for respective viral antigens in the MMIA) using a commercially available serological assay for each specific viral antigen, or an in-house ELISA. For CCHFV, MMIA-positive sera were assayed by ELISA (D-5052 VectoCrimea-CHF-IgG, Vector-Best) for anti-CCHFV NP IgG antibodies according to the manufacturer’s instructions. For RVFV and DOBV, MMIA-positive sera were assayed for anti-RVFV (FI 280a-1005 G, EUROIMMUN) or anti-DOBV (FI 278h-1010-1 G, EUROIMMUN) IgG antibodies by indirect immunofluorescence test (IIFT) according to the manufacturer’s instructions. The presence of IgG antibodies targeting MARV NP and EBOV NP in human sera was confirmed by in-house ELISAs. Briefly, the ELISA assay was developed using recombinant EBOV NP and MARV NP. 96-well plates were coated overnight with 1 μg/mL (50 ng/well) MARV NP and 0.7 μg/ml (35 ng/well) EBOV NP diluted in Carbonate buffer at 4°C. After washing in wash buffer (0.1% Tween-20 in PBS pH 7.2), blocking buffer (wash buffer containing 3% skim milk powder) was added to each well and incubated for 1 hour at room temperature. After the removal of the blocking buffer, serum samples diluted 1:400 in wash buffer containing 4% skim milk powder were added to each well in triplicate. Following a 1 hour incubation step at room temperature, serum samples were removed, and wells were washed 4 times in wash buffer prior to the addition of HRP conjugated goat anti-human secondary antibody (62–8420, Thermo Fisher Scientific). After 1 hour incubation, the secondary antibody was removed and cells were washed 8 times in wash buffer. TMB solution (S-004-3-TMB, Seramun) was then added to each well, and after a 15 minute incubation at room temperature 0.25 M H_2_SO_4_ solution (319570-500ML, Sigma Aldrich) was also added to each well and the plate was read in a Tecan Photometer analyser at a wavelength of 450 nm (referenced to 620 nm).

### Data processing and analysis

All data analysis was performed using R (version 3.5.1) [45] and GraphPad Prism (version 7.04). To this aim, median fluorescence intensity (MFI) readings from three technical replicates per sample measured by a Bio-Plex 200 (Bio-Rad) instrument were imported to R using the package XLconnect [46] and readxl [47]. Data processing was done using packages contained in the tidyverse [48] while plots were generated using ggplot2 [49] and ggpubr [50]. To determine the cutoff, the 99^th^ percentile for MFI values in the German blood bank population was determined for each antigen using the dlookr [51] package. Based on these cutoff values, contingency tables for both the German blood bank panel and the Guinea serum panel were prepared and Fisher's exact test was employed using GraphPad Prism to calculate whether or not statistically significant differences in the seroprevalences, and at which odds ratios, existed between the German and the Guinea sample panels.

## Results

### Optimisation of protein coupling and assay conditions

MMIA conditions were optimised using hyperimmune mouse and sheep sera and presumed negative human sera, in order to obtain the highest median fluorescent intensity (MFI) signal possible from specific antibodies present in serum samples, whilst maintaining the lowest possible background signal from non-specific interactions with antigen-coupled microspheres (S2 Fig). Assay optimisation should facilitate differentiation between positive and negative results and increase confidence in true positive and true negative results. The signal intensity of the MMIA was optimized by varying the amount of each NP bound to microspheres while serum titration, the usage of a specific buffer (LowCross) and the addition of *E*.*coli* lysate in a pre-incubation step was used to minimize non-specific background binding (S2 Fig). After optimisation signal intensities were found to be highest when employing 20 μg DOBV NP, 20 μg CCHFV NP, 35 μg RVFV NP, 20 μg MARV NP and 20 μg EBOV NP, respectively, per 1 × 10^6^ microspheres during protein coupling. Optimal assay conditions were achieved when using a 1:400 dilution of human sera and when a 30 minute pre-incubation step in assay buffer containing 5% *E*.*coli* lysate was included, in order to reduce/eliminate background MFI signals arising from antibodies targeting bacterial proteins present in human or animal serum (S2 Fig).

### MMIA validation using sera from virus-infected non-human primates or previously characterised human samples

**Fig 1:**
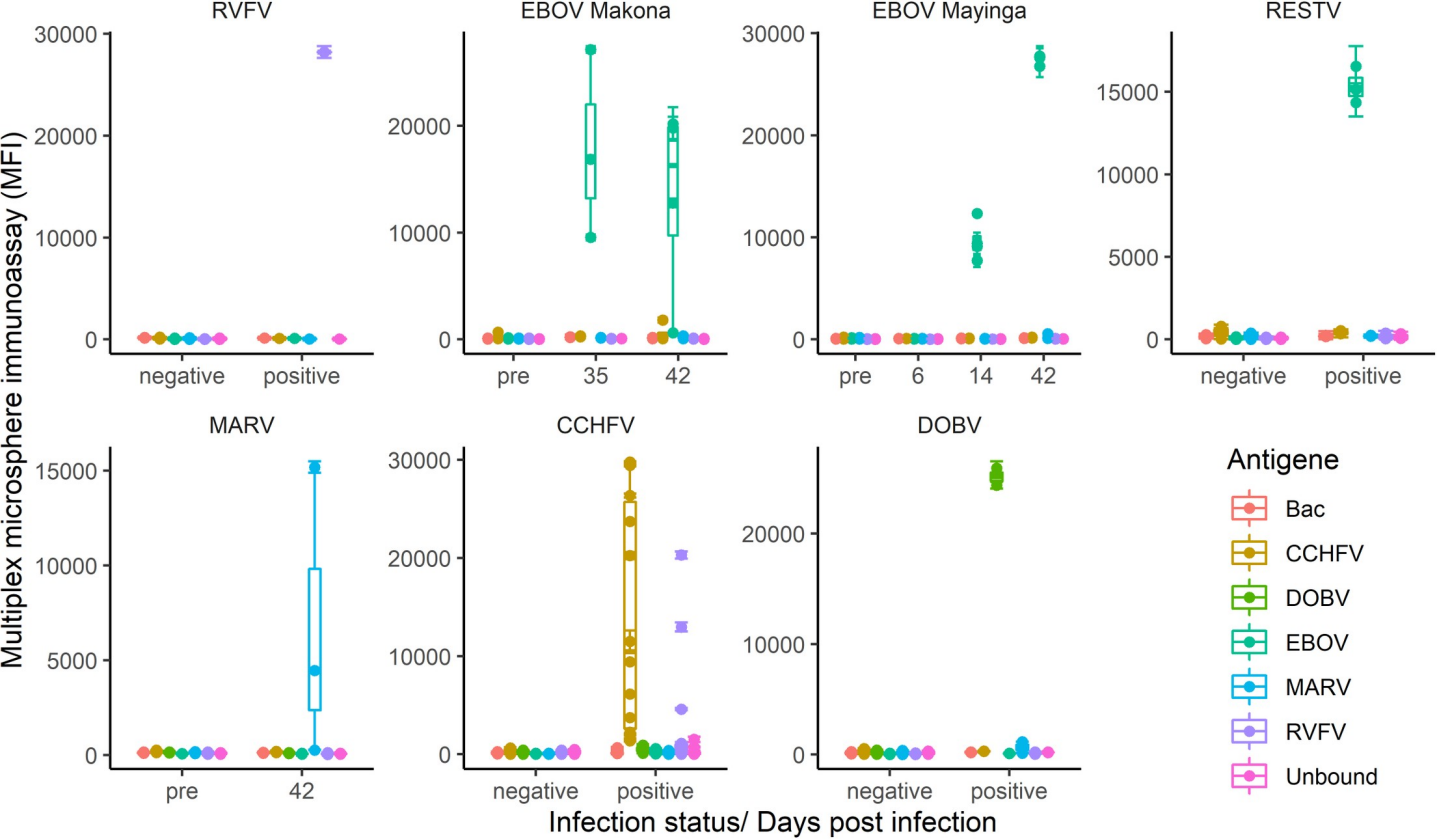
Validation of the MMIA using serum samples from humans or NHPs known to be infected with RVFV (1 NHP), EBOV (14 NHPs), MARV (3 NHPs), CCHFV (10 humans from Tajikistan or Nigeria), or DOBV (2 humans from Europe). Previously characterised serum samples were used as a validation panel to confirm the ability of the MMIA to detect anti-NP antibodies generated in response to infection with these viruses. These serum samples were assayed by MMIA under the same conditions that were used to assay the serum panels from the German blood bank and Guinea, thus providing a range of MFI values for each viral NP coupled microsphere region for known positive serum samples. For EBOV and MARV validation, serum taken from each NHP prior to experimental infection (pre) is compared to serum taken at various time points post-infection. For the RVFV NHP serum and for CCHFV and DOBV human serum, samples taken at a single time point after infection are compared to presumed negative human serum samples.

The ability of the MMIA to detect IgG antibodies specific for RVFV NP, EBOV NP and MARV NP, arising as a result of viral infection rather than protein inoculation, was verified using sera from NHPs experimentally infected with RVFV, EBOV or MARV (Fig 1). Here, testing of one sample from a NHP which had been experimentally infected with RVFV resulted in very high MFI readings (27,750) from the RVFV-NP-coupled microspheres, and MFI readings equivalent to background levels from the other viral NP-coupled microspheres, hereby confirming the specificity of our assay for detection of past RVFV infections. Serum samples from 3 NHPs infected with EBOV-Mayinga [39], 8 NHPs infected with EBOV-Makona [40,41] and 3 NHPs infected with RESTV taken at various time points post-infection (6, 14, 28, 35 or 42 days after infection) were compared in the MMIA with sera taken pre-infection (Fig 1). Here, the MMIA revealed an incremental increase in MFI readings of these sera from the EBOV NP-coupled microspheres. While the pre-bleed sera and the sera taken 6 days after infection showed MFI reading at background levels, MFI values increased to ~10,000 14 days after infection, then up to almost 30,000 42 days post infection. Simultaneously, signals equivalent to background levels were detected in these serum samples from the other viral NP-coupled microsphere regions, thus validating this assay for the quantitative detection of NP-specific IgG antibodies from EBOV-infected NHPs (Fig 1). Additionally, high MFI readings (~15,000) from RESTV infected NHP serum samples demonstrated the ability of the MMIA to detect evidence of infection from a range of ebolavirus species. Sera taken from 2 NHPs infected with MARV-Angola 42 days after infection resulted in high MFI readings in the MMIA from MARV NP-coupled microspheres when compared to the pre-infected sera. Serum from a third NHP taken 42 days after infection with MARV-Angola failed to show evidence of seroconversion in the MMIA, however, the confirmatory MARV-NP ELISA showed congruent results indicating that the MMIA faithfully reflects the serostatus of the infected animals (Table 1). The absence of a positive signal in the MMIA and ELISA is therefore most likely due to the absence of anti-MARV NP-specific IgG antibodies in the serum sample. Overall, these data validate the MMIA for the detection of anti-MARV NP-specific IgG antibodies in NHP sera.

**Table 1:** Comparison between the MMIA and the commercial IIFT/ELISA or in-house ELISA results using the serum samples from the validation panels. * Only validation panels where all samples did not test 100% positive were confirmed by commercial immunoassay. ** The same serum samples tested positive in both immunoassays.

		Serostatus (number positive/number tested)	
Samples	Antigen	MMIA	IFFT/ELISA
**CCHFV validation panel**	CCHFV	9/10**	9/10**
	RVFV	2/2	2/2
**DOBV validation panel**	DOBV	2/2	N/D*
**EBOV validation panel**	EBOV	17/33**	17/33**
**MARV validation panel**	MARV	2/6**	2/6**
**RVFV validation panel**	RVFV	1/1	N/D*

Next, a panel of 10 human serum samples that had previously been characterized for CCHFV serologically and by RT-PCR were used to validate the MMIA for anti-CCHFV NP antibody detection. All sera that had tested positive by CCHFV ELISA (Vector-Best, antigen CCHFV NP) also tested positive in the MMIA with MFI readings ranging from 3000 to 30,000 from the CCHFV NP coupled microspheres, thus confirming the ability of the MMIA to detect IgG antibodies generated during infection against CCHFV. Interestingly, 3 of these samples from Nigeria also had high MFI values from the RVFV NP coupled microspheres (MFI 4606, 12,786 and 20,233), potentially corresponding to human sera double positive for an anti-CCHFV and anti-RVFV NP titer.

Finally, the ability of the MMIA to detect anti-DOBV NP IgG antibodies in human sera was assayed using human serum samples from patients that had previously tested PCR/ELISA positive for anti-DOBV IgG. These serum samples were also positive in the MMIA, with high MFI readings (>20,000) detected from the DOBV NP coupled microspheres, and background level signals detected for the other viral NP coupled microsphere regions.

Overall, high MFI values (ranging from 3000–30,000 MFI units) were obtained from sera of known positive status for the corresponding viral NP-coupled microspheres, whilst the background MFI signal from the other 4 viral NP-coupled microspheres and the unbound microspheres remained low/negative (ranging from ~ 30–1800 MFI units).

### Establishment of a negative/positive threshold for each NP-coupled microsphere region

**Fig 2:**
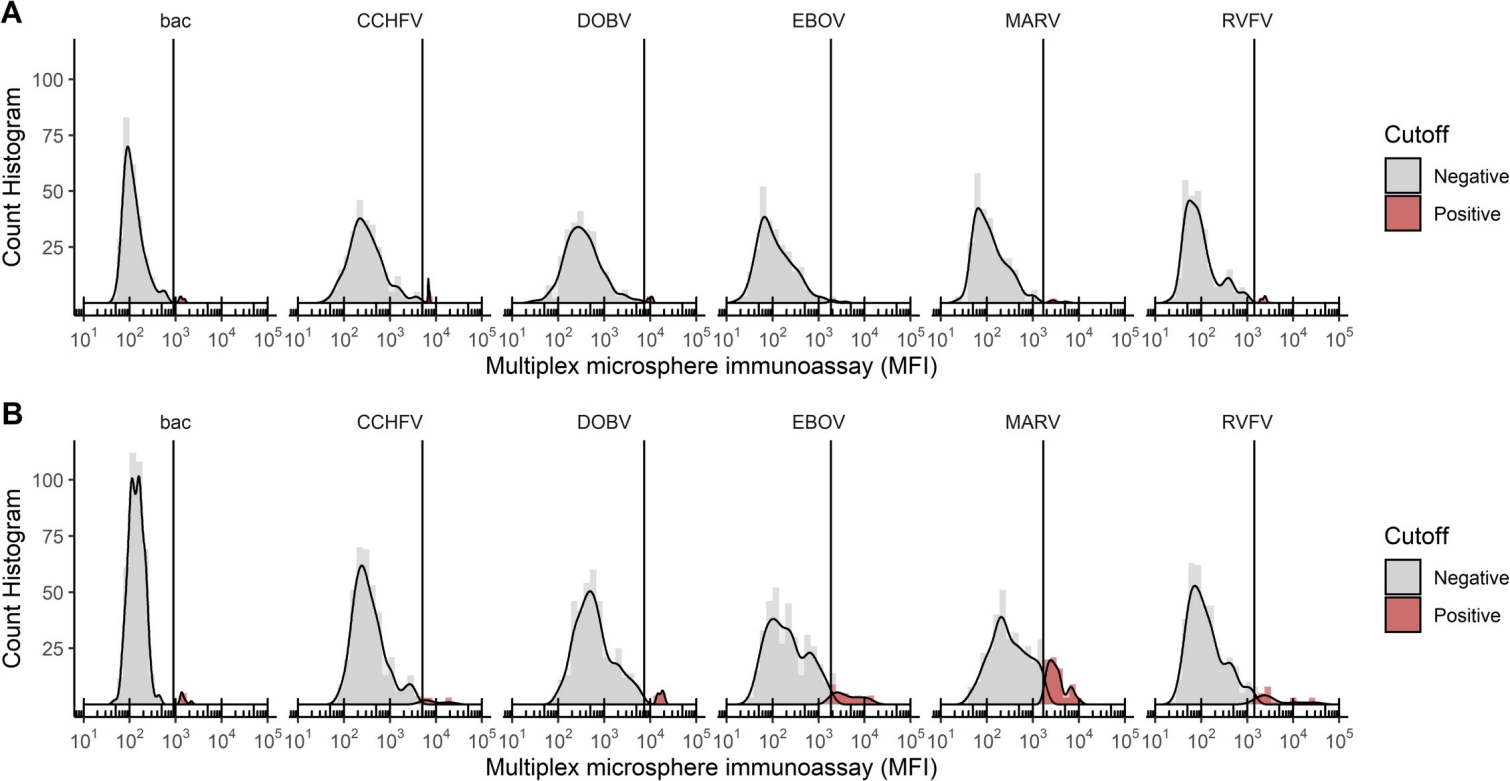
Histograms displaying the results of the MMIA for each viral NP. The x-axis displays the average MFI value for each serum sample tested in triplicate and the y-axis displays the number of serum samples that resulted in each MFI value. The serum panel from the (A) German blood bank and (B) from Guinea are shown individually. A conservative cutoff value is indicated by a vertical line on each plot, and was chosen based on the 99th percentile for serum samples in the German blood bank panel. This is a population based cut-off and assumes that 99% of serum samples in the German blood bank are negative for the indicated virus. Samples were assigned a positive or negative status based on this cutoff, and in all cases, negative serum samples are shown in grey and positive serum samples are shown in red.

In order to establish the negative/positive threshold for the MMIA, a panel of 88 human serum samples from the German blood bank was tested in triplicate. This resulted in different average MFI readings, and clustering pattern of MFI values, for each NP-bound microsphere region (Fig 2). For each microsphere region, the negative/positive threshold was then assigned as the 99^th^ percentile. This was based on the assumption that, with the exception of DOBV, none of the analysed highly pathogenic viruses are endemic in Germany, which is why a population based cut-off value was established. However, to allow for single sera with higher reactivity the 99^th^ percentile was chosen to discern positive from negative sera. It should be noted that one serum sample from the German blood bank resulted in a strong signal from the DOBV NP-coupled microspheres.

### Screening of human serum samples collected from Guinea in 2011–2012 using the MMIA

Serum samples collected from febrile human patients in Guinea in 2011 and 2012 (129 serum samples) were screened for NP-specific IgG antibodies against CCHFV, RVFV, DOBV, EBOV and MARV using the MMIA. The MMIA was performed in triplicate and the MFI results for each antigen were analysed and compared with those obtained from the German blood bank sample set (Fig 2). Here, the overall distribution patterns of log-transformed MFI values were similar between the two serum sample sets. Samples were determined to be positive or negative for each viral NP based on the conservative negative/positive threshold (cutoff) value determined by using the 99^th^ percentile of the German blood bank samples. The total number of positive and negative serum samples for each viral NP in both populations (German blood bank and Guinea) are summarised and compared in Table 2.

**Table 2:** Comparison of the number of serum samples that tested positive and negative against each viral NP in the German blood bank and Guinean serum sample sets. The median MFI readings from three technical replicates per sample were analysed in R, and a positive or negative status was assigned to each serum sample. Positivity or negativity was based on a cutoff MFI value that was determined for each viral NP based on the 99^th^ percentile for MFI values in the German blood bank sample set. Statistically significant differences in seroprevalence rates between the two sample sets and odds ratios were calculated using Fisher’s exact test.

Group		Percentage		Frequency†		Cutoff	Fisher's exact test		
Antigen	Serostatus	German Blood donor	Guinea panel	German Blood donor	Guinea panel	MFI	P-value	Odds ratio	95% CI
Bacterial lysate	Negative	98.9	98.5	87	127		>0.9999	n.a.	
	Positive	1.1	1.6	1	2	896.4			
CCHFV	Negative	99.6	99.0	87	128		>0.9999	n.a.	
	Positive	0.4	1.0	0	1	5112.0			
DOBV	Negative	98.9	97.7	87	126		0.65	n.a.	
	Positive	1.1	2.3	1	3	7389.1			
EBOV	Negative	98.9	91.5	87	118		0.03	8.11	1.4 to 88,5
	Positive	1.1	8.5	1	11	1866.5			
MARV	Negative	98.9	81.4	87	105		<0.0001	11.33	2.3 to 64.1
	Positive	1.1	18.6	1	24	1689.7			
RVFV	Negative	98.9	94.1	87	121		0.09	n.a.	
	Positive	1.1	5.9	1	8	1433.0			

† Only complete cases (all three replicates either positive or negative) were analysed

A statistically significant difference between the number of positive serum samples in the sample set from Guinea and the German blood bank was only observed when comparing reactivity against EBOV NP and MARV NP coupled microspheres (Table 2). In both cases, a statistically significantly larger number of samples reacted positively against EBOV NP and MARV NP in the serum set from Guinea compared to the serum set from the German blood bank. Indeed, this analysis revealed that 18.6% of the serum samples within the Guinean sample set were potentially positive for MARV NP antibodies and 8.5% were potentially positive for EBOV NP antibodies (Table 2). However, false positive readings, cross reactivity or higher background readings for individual serum samples also cannot be ruled out at this stage. In addition, several serum samples also reacted positively in the MMIA against other viral NPs in the sample set from Guinea, especially against RVFV NP coupled microspheres (Table 2). However, the overall reactivity for most sera was lower (below 10,000 MFI) as compared to the infected non-human primates or human samples with known infections status (Fig 1), most likely indicating an absence of recent contact with the highly pathogenic viruses tested.

### Comparison of the MMIA with commercial or in-house immunoassays

In order to confirm the results of the MMIA, positive serum samples from the sample set used for assay validation, the German Blood bank donors and the Guinean sample sets were tested using the corresponding commercially available immunoassay, or in the case of EBOV and MARV, in-house ELISA. The commercial immunoassays used were as follows: CCHFV (NP-based ELISA from VectorBest), RVFV (IIFT using RVFV infected cells from EUROIMMUN) and DOBV (IIFT using DOBV infected cells from EUROIMMUN). Here, all highly positive samples from the MMIA validation sample set (Fig 1) were confirmed as positive by the commercial assays for their respective viral NPs (Table 1). In addition, two of the serum samples that were part of the CCHFV validation panel that had high MFI readings for the RVFV NP-coupled microspheres (12,786 and 20,233), also tested positive in the RVFV IIFT (titers of 1:400 and 1:1600 respectively). The serum sample from the German blood bank that had a high MFI reading from the DOBV NP-coupled microspheres (9916) also tested positive in the DOBV IIFT (titre of 1:800) thus confirming the accuracy of the MMIA. Two of the possible RVFV positive samples in the Guinean sample set (MFI values 28,876 and 10,751), were tested using the commercial RVFV IIFT, however only one (MFI 28,876) was confirmed positive (titer of 1:1,600). This might be due to the increased sensitivity of the MMIA in comparison to the IIFT. Probably for the same reason, three samples from Guinea that showed a relatively strong signal from the DOBV NP coupled microspheres tested negative by IIFT. Another reason could be differences in the antigens used in these assays (the antigens in the IIFT are derived from DOBV-infected cells, whereas the antigen in the MMIA is recombinant DOBV NP) and virus strains circulating in Guinea. A single sample from the Guinean sample set had MFI readings >10,000 for CCHFV NP-coupled microspheres, and this sample also had a positive result when tested by the commercial CCHFV ELISA. It should be noted that ELISAs are generally more sensitive than IIFTs.

**Fig 3:**
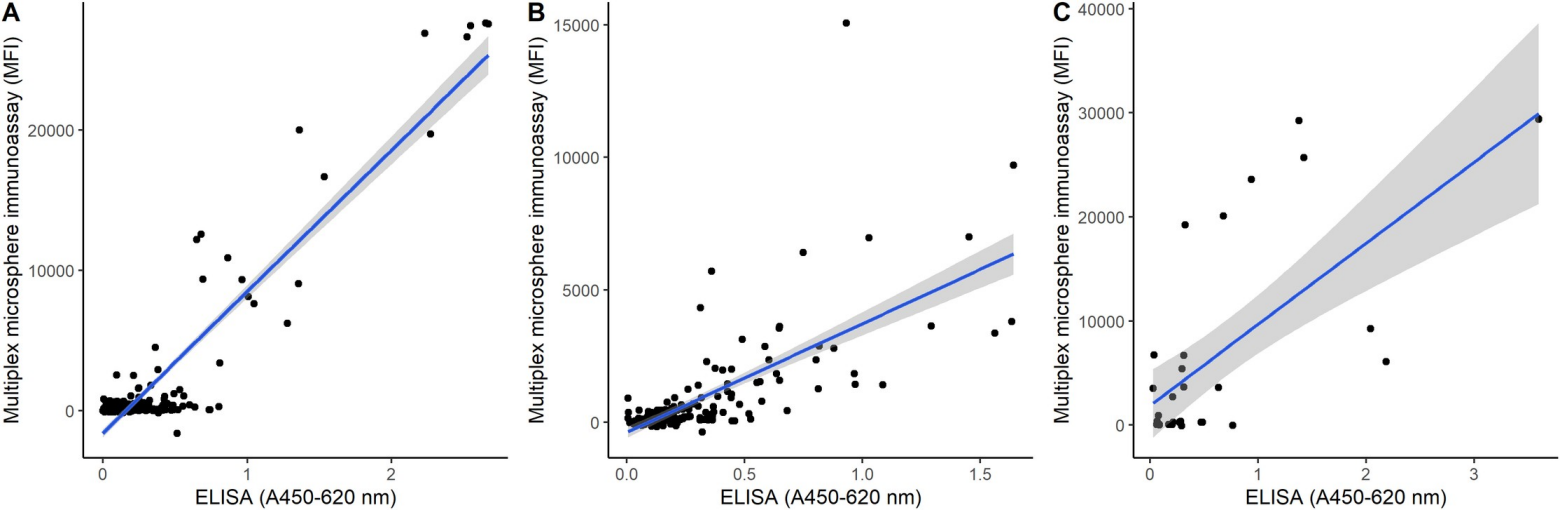
Correlation between MMIA results and commercially available or in-house ELISAs for EBOV (A), MARV (B) and CCHFV (C). For both the MMIA and the ELISAs results were obtained from 3 technical replicates for each sample and the average MFI value for each NP (EBOV, MARV or CCHFV) was then plotted against the average absorbance values obtained from the same sample in the corresponding ELISA. In (A) and (B) all serum samples from the Guinea panel and the validation panel were tested by in-house EBOV or MARV ELISAs, in (C) a subset of samples from the Guinea panel and validation panel were tested by commercial CCHFV IgG ELISA.

Further analysis using more samples from the Guinean sample set together with the validation sample set revealed a positive correlation between the CCHFV ELISA and MMIA results (Fig 3C). In the case of EBOV and MARV, commercial immunoassays were not available, therefore in-house ELISAs were developed using recombinant EBOV NP and MARV NP. Comparison of the results from the MMIA and in-house EBOV and MARV ELISAs revealed a tight positive correlation between the two techniques (Fig 3A and 3B). Overall the agreement between the ELISA/IIFT data and the MMIA data supports the use of the MMIA as a multiplex screening tool for the detection of IgG antibodies against the NPs of CCHFV, RVFV, DOBV, EBOV and MARV.

### Adaptation of the MMIA to analyse bat serum samples for antibodies targeting EBOV and MARV

**Fig 4:**
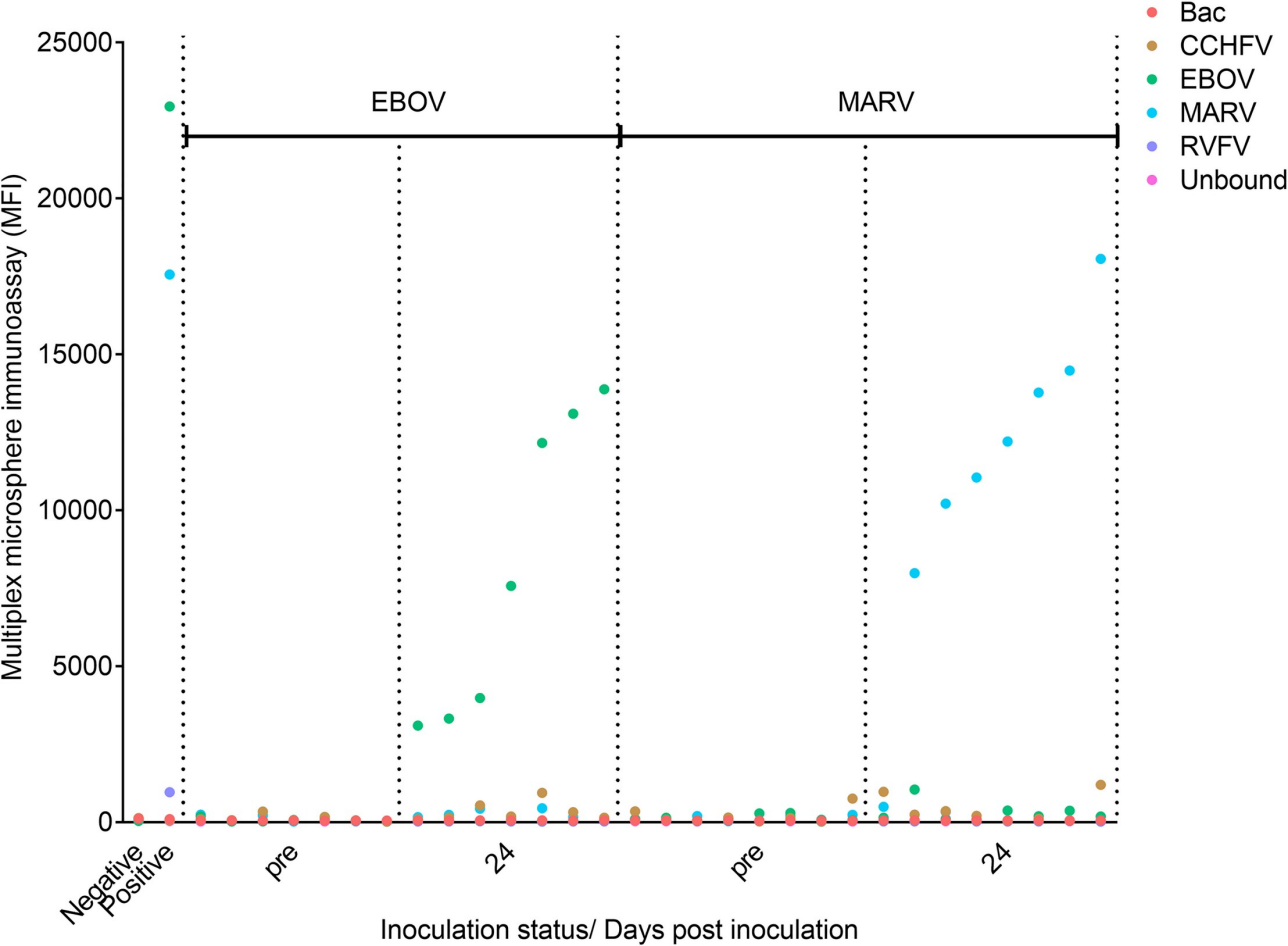
Analysis by MMIA of serum samples taken pre- and post-inoculation with EBOV- and MARV-VLPs from *Mops condylurus* micro bats. Serum samples were taken from micro bats 24 days after inoculation with EBOV VLPs and MARV VLPs, and their reactivity to EBOV NP and MARV NP coupled microspheres was compared to sera taken pre-inoculation. The MMIA was adapted to detect bat antibodies by exchanging the SAPE-conjugated anti-human secondary antibody with SAPE-conjugated protein A and protein G. MFI readings for the same negative and EBOV-NP and MARV-NP positive NHP sera used as controls in all MMIAs are shown in the first 2 columns.

The MMIA was adapted to detect specific antibodies targeting EBOV NP and MARV NP from bat serum samples by using a mixture of biotinylated protein A and protein G in place of the biotinylated anti-human IgG secondary antibody. The sera analysed included sera taken pre-inoculation, as well as sera taken 24 days after the inoculation of *Mops condylurus* micro bats with either EBOV- or MARV-VLPs. Pre- and post-inoculation sera were analysed from 7 bats inoculated with EBOV-VLPs and 8 bats inoculated with MARV-VLPs. When analysed by MMIA the sera taken from bats 24 days after inoculation with EBOV- or MARV-VLPs showed high MFI signals (MFI values between 2000–15,000) from their corresponding NP-coupled microsphere regions, in contrast to MFI signals corresponding to background levels (MFI values < 500) from the other 3 viral NP-coupled microsphere regions and from the pre-inoculation bat sera (Fig 4). Only a single bat inoculated with MARV-VLPs failed to seroconvert. These data demonstrate that this MMIA could be effectively adapted to detect NP-specific EBOV and MARV IgG antibodies from bat sera, and, thus, underscores the versatility of this assay.

## Discussion

Monitoring of highly pathogenic viruses in human and wildlife populations is critical to enable the effective assessment of the risk of spill-over human infectious disease outbreaks, as well as epizootics. Furthermore, knowledge of circulating viruses in certain areas aids differential diagnosis decisions of local healthcare providers for febrile patients. In this study, we describe the development of a broadly reactive MMIA which detects IgG antibodies targeting the NPs of CCHFV, DOBV, RVFV, EBOV and MARV and that could be used as a screening tool in seroprevalence studies to assess the prevalence of antibodies against these viruses in certain human and animal populations. By targeting IgG instead of IgM antibodies our MMIA is designed to detect evidence of past infection with these viruses, not recent infection. IgG antibodies can still circulate in serum for years after infection with a specific virus, thereby increasing their chances of being detected, whereas IgM antibodies only circulate for a few months post-infection, reducing the window of opportunity for detection. Therefore when surveilling a novel cohort of humans or animals, an IgG based assay such as our MMIA can be used to assess whether these viruses have previously circulated in a certain region, then further studies of seropositive populations could include specific IgM detection assays to look for evidence of more recent infections.

MMIAs have several advantages over ELISA and IIFT as they enable the simultaneous detection of antibodies against multiple target antigens in a single assay, thereby conserving serum samples, reagents and time [44,52]. In addition MMIAs have been shown to be more sensitive and more cost effective than ELISAs [24,53,54]. However, the specific equipment required to perform the MMIA and the initial associated start-up cost may present a barrier to starting to work with these assays, and the higher sensitivity of the MMIAs compared to other immunoassays can present significant challenges when developing novel assays and defining a positive/negative cutoff and evaluating low positive samples. The lack of a standard, uniform immunoassay to confirm the results of novel MMIAs further complicates their evaluation and the designation of a cutoff value. For example, in this study both ELISAs and IIFTs (which are known to have different sensitivities), commercial as well as those designed in-house, were used to compare and confirm the results of the MMIA, as there was not one standard format of commercial immunoassay available. This made the interpretation of the results more challenging than if, for example, ELISAs of comparative sensitivities existed for the detection of IgG antibodies against each virus. There are several different statistical methods that can be employed to define a cutoff value in novel MMIAs, but in the absence of definitive knowledge of previous infection, it may not be possible to satisfactorily resolve individual samples with MFI values very close to the cutoff. MMIAs of various different formats have been developed to detect antibodies against numerous viral pathogens including multiple arboviruses [55,56] and highly pathogenic viruses such as EBOV, Lassa virus, RVFV, CCHFV, and Hendra and Nipah viruses [53,54,57,58]. An EBOV and LASV MMIA developed using both native antigen from infected cells and recombinant antigen from a transfected human cell line (glycoprotein complex (GPC) and nucleocapsid protein (NP) from LASV and glycoprotein (GP) and viral matrix protein 40 (VP40) from EBOV) was shown to be 5 and 25 times more sensitive in detecting IgM antibodies against LASV and EBOV, respectively, compared to the equivalent ELISAs [54]. MMIAs which simultaneously detect antibodies against multiple recombinantly expressed viral proteins of either RVFV or EBOV [53,58] have been developed. MMIAs such as these not only provide greater certainty of a true positive result though the detection of antibodies against multiple antigens of the same virus, but also enable the possibility of differentiation between vaccinated and infected individuals in a single assay [59,60].

We chose to use the recombinant, bacterially expressed NPs of CCHFV, DOBV, RVFV, EBOV and MARV as the viral antigen to couple to microspheres. The NPs of these viruses have previously been shown to be highly immunogenic, and are often conserved between viral species of the same genus [61,62] enabling the detection of antibodies targeting a broad range of virus species in a single assay. Indeed, the successful detection of anti-EBOV NP antibodies generated as a response to infection by different ebolaviruses, EBOV (Mayinga and Makona) and RESTV in our MMIA, highlights the broadly reactive nature of EBOV NP and validates our choice of NP as the MMIA antigen for this reason. Other viral proteins were also considered for use as the ‘bait’ antigen, for example the viral glycoproteins and non-structural proteins (NS or virion proteins). However, these proteins have been shown to be less immunogenic than NP or less broadly reactive than NP in previous studies [58,63,64] and, therefore, were not chosen.

The use of recombinant bacterially expressed NPs as the antigen in the MMIA negates the necessity for BSL-4 facilities to grow these highly pathogenic viruses to use as ‘bait’. However, due to the bacterial expression of the NPs, high background MFI signals were detected against most viral antigen-coupled microsphere regions when the MMIA was tested using human control sera. These signals presumably arose as a result of the presence of contaminating bacterial proteins co-purified with the viral NPs that were co-coupled to the microspheres, reacting with anti-*E*.*coli* antibodies present in human sera. This high background was effectively eliminated by the pre-incubation of each serum sample with assay buffer containing 5% *E*.*coli* lysate. It is thought that during this incubation, anti-*E*.*coli* antibodies present in the serum bound to their target proteins, thus removing them from the pool of available antibodies that could bind to the bacterial proteins coupled to the microspheres during the assay. This has previously been shown to be an effective technique to reduce or remove background signals from MMIAs [65]. In addition, one microsphere region was covalently coupled to *E*.*coli* cell lysate and acted as a control during the assays–any serum samples that had MFI readings above background levels to this microsphere region would be discounted from further analysis.

Following assay optimisation, stringent assay conditions as well as stringent positive/negative cutoff values were chosen in order to decrease the chance of false positive signals. Given the highly sensitive nature of MMIAs in comparison to ELISAs [54] and IIFTs, it was thought that low positive MFI signals might not be able to be verified by an alternative serological assay, therefore, stringent assay conditions were chosen to reduce the likelihood of this situation occurring. Indeed, in our MMIA the majority of serum samples that resulted in a very high MFI value (> 20,000) for a specific viral antigen were confirmed as positive using either the corresponding ELISA or IIFT. However, some samples that had a clearly higher than background MFI value (between 4000 and 12,000 in the case of RVFV NP-coupled microspheres, for example), sometimes did not test positive by an alternative immunoassay, presumably due to the higher sensitivity of the MMIA. Samples with a low positive MFI signal such as these must be considered on an individual basis, as they could also possibly be false positives, arising for example, due to incorrect storage of serum samples and sample degradation. Titration of such samples and re-testing in the MMIA may provide more clarity, as often false positive samples will test negative at a lower dilution than true positive samples. However, it should also be noted that some very low positive signals may not be detected in our MMIA due to the stringent assay conditions that were chosen.

A set of 129 serum samples collected in Guinea in 2011 and 2012 were assayed in the MMIA to further test the MMIA and to screen this population for IgG antibodies against CCHFV, RVFV, DOBV, EBOV and MARV NPs. Any serum that resulted in high MFI values was also tested using the commercially available immunoassay, or in the case of EBOV and MARV, by in-house ELISAs. Overall, there was a positive correlation between the MMIA and the alternative ELISA or IIFT, indicating the MMIA functioned well as an IgG antibody screening tool. However, the commercial immunoassays that were used also have their limitations, and the samples that tested positive using the in-house and commercial ELISAs were not titrated. Therefore, further development and validation of this MMIA could include additional testing of positive samples using different assays by a different laboratory, and titration and re-testing in the ELISA and MMIA. This might provide more of an indication of whether these sera were reacting specifically to the viral NPs, as well as potentially providing more information about the relative sensitivity of the 2 assays.

However, there were 3 serum samples from the Guinean sample set that tested positive for DOBV NP by MMIA but were negative in the corresponding IIFT. These could be false positives, or the results may be due to the fact that the slides of the DOBV confirmatory IIFT (EUROIMMUN) are made using DOBV-infected cells whilst the MMIA uses the more broadly reactive DOBV NP as the antigen. The presence of DOBV itself has yet to be documented on the African continent, however other novel Hantaviruses have been isolated and sera were shown to cross react with DOBV [13,16,66]. Therefore, circulating hantavirus strains in Guinea may elicit an antibody reaction that can be detected by the more cross-reactive NP-coupled to microspheres, but these antibodies do not result in a detectable reaction when assayed using DOBV-infected cells.

There were serum samples that were reactive against more than one viral antigen in the MMIA, corresponding to human sera double or triple positive for EBOV, MARV, RVFV, DOBV or CCHFV NPs. It is conceivable that a sample could test positive against more than one of these viral antigens (indeed, 2 serum samples from the CCHFV validation panel were independently confirmed by commercial IIFT to also potentially be positive for RVFV IgG) due to the individual having been infected with more than one of these viruses. However it is also possible that some serum samples are more non-specifically reactive than others, and may produce false positive results. Another MMIA could be developed by coupling other antigens from these viruses to microspheres (glycoproteins or matrix proteins for example) in order to increase assay specificity, after the initial screen had been completed with our broadly reactive NP based MMIA. Serum samples have, for example, tested positive against the NP of EBOV, but tested negative against the EBOV glycoprotein [67]. It is speculated that sera such as this could be reacting against the broadly reactive NP of distantly related filoviruses. Interestingly, when the MMIA results of the sample set from Guinea were compared with those of the German blood bank, only the number of positive reactions against the EBOV NP and MARV NP were found to be increased to a statistically significant degree in the sample set from Guinea. This implies the possible circulation of filoviruses in this human population before 2013 (samples were collected in 2011 and 2012). However, assays to more clearly determine specificity to EBOV and MARV (for example serological assays using other EBOV and MARV antigens, such as the glycoprotein or non structural proteins as bait, or titration of the serum samples) should be performed before any definitive interpretation of these results are made. These data are however in accordance with previous serological surveys of human samples for antibodies against EBOV and MARV: high seropositive rates have been reported for EBOV and MARV in human populations in the Central African Republic, the Republic of Congo and the Democratic Republic of the Congo [68–70]. Indeed, an overall seroprevalence rate of 15.3% for antibodies against EBOV has previously been reported in Gabon [71]). Also in agreement with our findings, a recent study using a MMIA specific for EBOV documented EBOV IgG antibodies in human serum samples collected in Guinea in 2012, although the first recorded case of EBOV in Guinea was not reported until the following year (December 2013) [67]. There is an increasing body of evidence that EBOV and MARV are capable of causing asymptomatic or mild infections, which may account for the detection of EBOV- and MARV-specific antibodies in the human population in the absence of a history of haemorrhagic symptoms or an outbreak of haemorrhagic disease [72–74]. Indeed recent studies have documented the presence of anti-EBOV antibodies in populations with no history of EBOV outbreaks in the Democratic Republic of the Congo (overall 11% positivity rate of IgG antibodies to Zaire Ebola virus) and Southwestern Uganda [75,76]. Alternatively, it is also possible that these antibody prevalence rates may be accounted for by milder, as-yet-undocumented strains/species of filoviruses that may be circulating undetected in human populations. In support of this theory, genomic sequences of 2 new filoviruses (Bombali virus and **Měnglà virus**) have recently been discovered in bats, although their ability to infected humans has yet to be determined [18,77]. Interestingly, serum from fruit bats in Southeast Asia was found to be reactive against the glycoprotein of various filoviruses when tested by MMIA [78], suggesting the presence of filoviruses antigenically related to Bundibugyo, Ebola, and Sudan virus in Southeast Asia, however the causative virus has yet to be identified. When human serum taken from individuals involved in hunting these species of bats in Northeast India were tested using the same MMIA, several human serum samples also reacted positively against these filovirus glycoproteins, as did serum samples taken from the bats they hunted [79]. This implies zoonotic spillover events had occurred, in the absence of human disease outbreaks being reported. These data support the possibility that filoviruses related to EBOV and MARV may circulate un-noticed in human populations.

Indeed, our MMIA was also adapted to detect antibodies from bats, *Mops condylurus*, after inoculation with EBOV- and MARV-VLPs. High MFI readings were obtained 24 days post inoculation from EBOV NP and MARV NP-coupled microspheres, confirming an antibody response from the micro bats, and demonstrating the adaptability of the MMIA. In fact, this assay could easily be adapted to screen animal sera from other species for the presence of antibodies against these viruses, to confirm their circulation in defined animal populations, such as livestock. CCHFV, RVFV, DOBV, EBOV and MARV are all zoonotic pathogens that are transmitted from animals or insect vectors to humans, and knowledge regarding their presence and circulation in animal populations could be invaluable to help identify at-risk human populations that live in close proximity to these animals, as well as helping to protect the animals themselves. Identification of livestock populations with exposure to CCHFV and RVFV, for example, would imply these viruses are circulating in the region these livestock are kept, and indicate vector control measures should be strictly adhered to or implemented, to reduce the risk of human exposure to these viruses. In case of evidence of CCHFV circulation in livestock, slaughter house workers should take precautions to avoid virus exposure. In addition the MMIA could be used to assay wild animal populations to identify zoonotic virus reservoirs. The MMIA would enable the high throughput detection of antibodies against several viruses simultaneously using a very small sample volume, which would be advantageous over other methods when, for example, surveilling small mammals or birds where humane extraction of large quantities of serum may not be feasible.

## Supporting information

S1 Fig Coomassie stained gels of purified recombinant NPs of EBOV, MARV, CCHFV, RVFV and DOBV. ~6 ug viral NP was loaded per lane of a polyacrylamide SDS gel (with the exception of DOBV NP). Following electrophoresis at 200 V for 30 minutes, gels were stained by overnight incubation at room temperature in Coomassie stain. After de-staining, gel images of were captured using a ChemiDoc imager. The dominant band in each gel image corresponds to the correct molecular weight for the indicated viral NPs.
(TIF)

S2 Fig MMIA optimisation. The MMIA was optimised by varying the amount of each purified NP that was bound to 1 x 10^6^ microspheres in order to achieve the maximum MFI signal intensity, whilst maintaining the lowest possible background MFI signal. (A) depicts the optimisation of the amount of CCHFV NP coupled to microspheres using 5 μg, 20 μg and 50 μg of CCHFV NP per 1 x 10^6^ microspheres. CCHFV NP binding was detected using dilutions of hyper-immune sheep sera ranging from 1:12,800 to 1:640,000 diluted in assay buffer. (B) depicts the optimisation of the amount of RVFV NP coupled to microspheres using 7 μg, 35 μg and 70 μg of RVFV NP per 1 x 10^6^ microspheres. RVFV NP binding was detected using dilutions of hyper-immune mouse sera ranging from 1:10,000 to 1:640,000 diluted in assay buffer. The MMIA was also optimised in order to reduce the MFI signals generated by antibodies in serum binding to bacterial proteins present in the purified NP preparations, by pre-incubating the serum in assay buffer containing 5% *E*.*coli* lysate for 30 minutes. In (C) the MFI signals arising from the protein coated and unbound microspheres after a 1 hr incubation with human serum diluted 1:100, 1:200 or 1:400 in assay buffer is shown. This is in contrast to (D) where the MFI signals arising from the same human serum samples are shown, however in (D) the serum dilutions were first pre-incubated in assay buffer containing 5% *E*.*coli* lysate, prior to the 1 hr incubation with protein coated and unbound microspheres. The horizontal dotted line in (C) and (D) represents an MFI signal of 300 units.
(TIF)
