# Supplementary figures and images for: Development of a multiplex microsphere immunoassay for the detection of antibodies against highly pathogenic viruses in human and animal serum samples
Source: PLoS Negl Trop Dis. 2020 Oct 23;14(10):e0008699. doi: 10.1371/journal.pntd.0008699 (PMC7641473; doi:10.1371/journal.pntd.0008699)

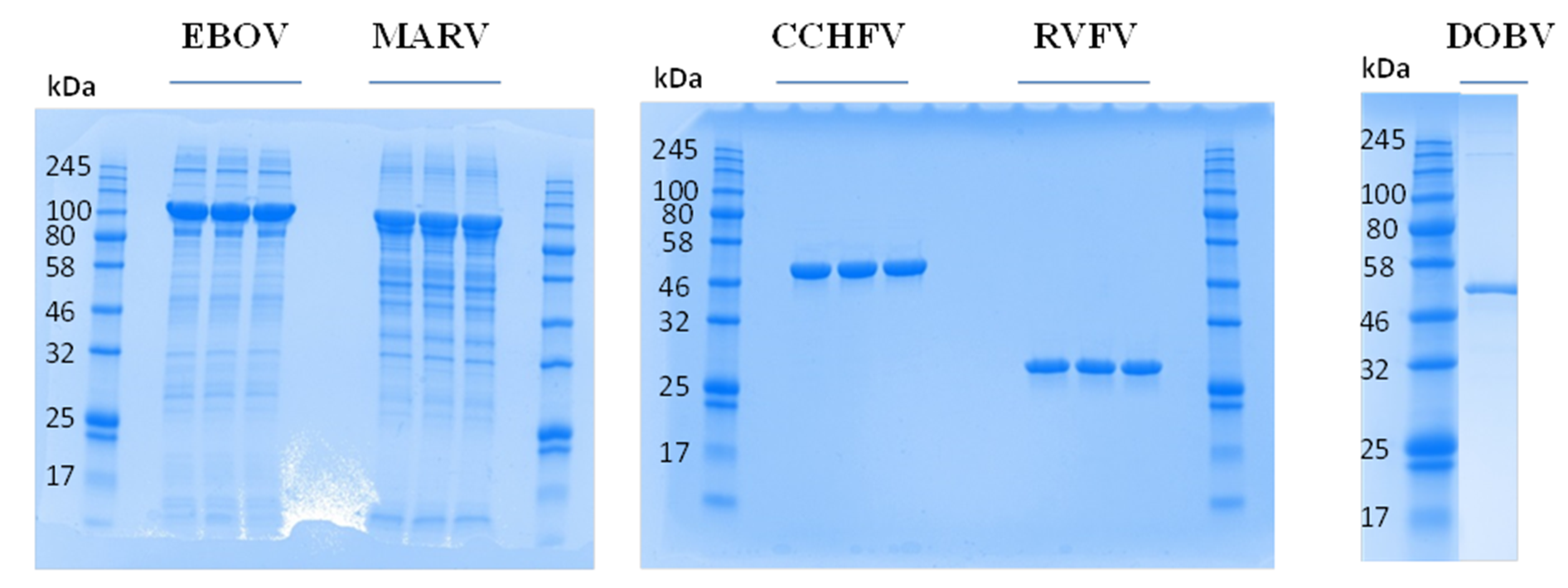

Supplement: S1 Fig — ~6 ug viral NP was loaded per lane of a polyacrylamide SDS gel (with the exception of DOBV NP). Following electrophoresis at 200 V for 30 minutes, gels were stained by overnight incubation at room temperature in Coomassie stain. After de-staining, gel images of were captured using a ChemiDoc imager. The dominant band in each gel image corresponds to the correct molecular weight for the indicated viral NPs. (TIF) [file pntd.0008699.s001.tif]

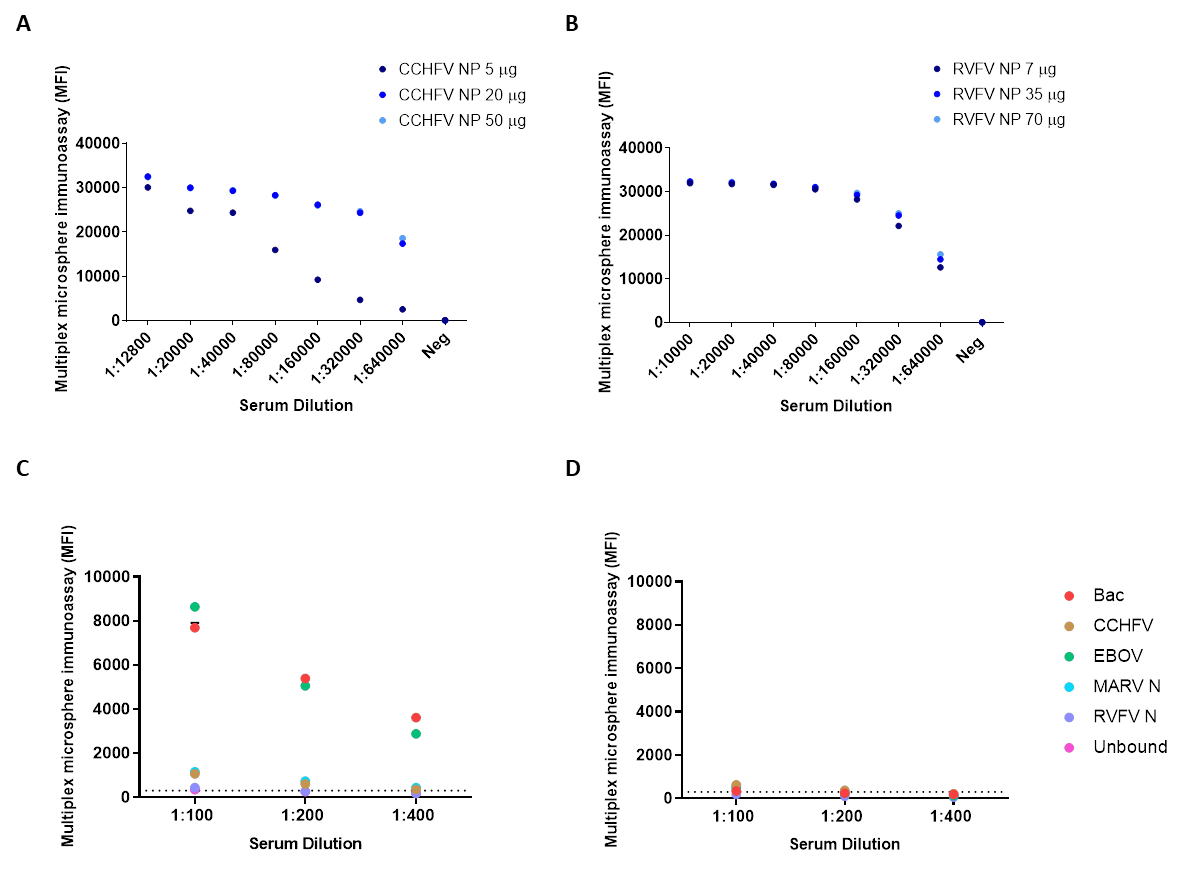

Supplement: S2 Fig — The MMIA was optimised by varying the amount of each purified NP that was bound to 1 x 10^6^ microspheres in order to achieve the maximum MFI signal intensity, whilst maintaining the lowest possible background MFI signal. (A) depicts the optimisation of the amount of CCHFV NP coupled to microspheres using 5 μg, 20 μg and 50 μg of CCHFV NP per 1 x 10^6^ microspheres. CCHFV NP binding was detected using dilutions of hyper-immune sheep sera ranging from 1:12,800 to 1:640,000 diluted in assay buffer. (B) depicts the optimisation of the amount of RVFV NP coupled to microspheres using 7 μg, 35 μg and 70 μg of RVFV NP per 1 x 10^6^ microspheres. RVFV NP binding was detected using dilutions of hyper-immune mouse sera ranging from 1:10,000 to 1:640,000 diluted in assay buffer. The MMIA was also optimised in order to reduce the MFI signals generated by antibodies in serum binding to bacterial proteins present in the purified NP preparations, by pre-incubating the serum in assay buffer containing 5% E.coli lysate for 30 minutes. In (C) the MFI signals arising from the protein coated and unbound microspheres after a 1 hr incubation with human serum diluted 1:100, 1:200 or 1:400 in assay buffer is shown. This is in contrast to (D) where the MFI signals arising from the same human serum samples are shown, however in (D) the serum dilutions were first pre-incubated in assay buffer containing 5% E.coli lysate, prior to the 1 hr incubation with protein coated and unbound microspheres. The horizontal dotted line in (C) and (D) represents an MFI signal of 300 units. (TIF) [file pntd.0008699.s002.tif]
